# Pressure gradient means flow

**DOI:** 10.1186/s13054-023-04442-5

**Published:** 2023-04-18

**Authors:** Jueyue Yan, Zhipeng Xu, Xujian He, Chenkan Chen, Tong Li

**Affiliations:** Department of Critical Care Medicine, The First Affiliated Hospital, Zhejiang University School of Medicine, No. 79 Qingchun Road, Hangzhou, 310003 Zhejiang Province China

Dear editor,

The indications for ECMO as temporary cardiopulmonary support are growing [1]. Peripheral vascular intubation is usually used in emergency situations [2]. Distal limb necrosis is a common complication of PVAECMO, with an incidence of 10–70% [3]. Indwelling distal perfusion cannula (DPC) is considered to be a common method to prevent ischemic necrosis of distal limbs [4]. However, in clinical practice, it is still difficult to ensure proper perfusion of the lower extremities even if the patient receives DPC indwelling. One of the important reasons for this is the lack of effective perfusion monitoring of the distal limb, which inevitably leads to hypoperfusion or luxury perfusion. The 1/4-inch tube often serves as a bridge connecting the perfusion tube to the DPC (Fig. 1). According to the famous Poiseuille’s law, *R* = 8*nL*/(*πr*^4^), which is *Q* = Δ*p*/*R*. Since the resistance properties of the pipeline will not change in a short time, the flow rate can be determined as long as the pressure difference on the pipeline is determined. Due to the characteristics of the DPC, it is difficult to measure the pressure change at both ends of the DPC in a non-invasive manner. The pressure gradient of the 1/4-inch connecting pipe can be selected to measure the flow through the DPC indirectly. Combined with the results of in vitro fluid experiments and clinical observations, under the assumption that the patient’s hemodynamic level and ECMO output power remain unchanged, the pressure at the ends of the 1/4-inch connecting tube is *P*1, *P*2 (the pressure near the perfusion tube is *P*1), Δ*P* = *P*1–*P*2; a decrease in both *P*1*P*2 and Δ*P* occurs when a pipeline obstruction event occurs before the 1/4-inch connection (Fig. 1A). When a conduit obstruction event occurred in the perfusion tube (Fig. 1C), *P*1, *P*2, and Δ*P* all increased. When a pipe-blocking event occurred in the 1/4-inch connection pipe (Fig. 1D), *P*1 is unchanged, *P*2 is decreased, and Δ*P* is increased. When a pipe occlusion event occurred after the 1/4-inch connection (Fig. 1E), *P*1 remained unchanged, *P*2 increased, and Δ*P* decreased. We suggest that routine collateral perfusion tubes for pressure monitoring may help to reduce the incidence of lower limb ischemia as well as to provide localization diagnostic evidence when lower-limb hypoperfusion occurs.

**Fig. 1:**
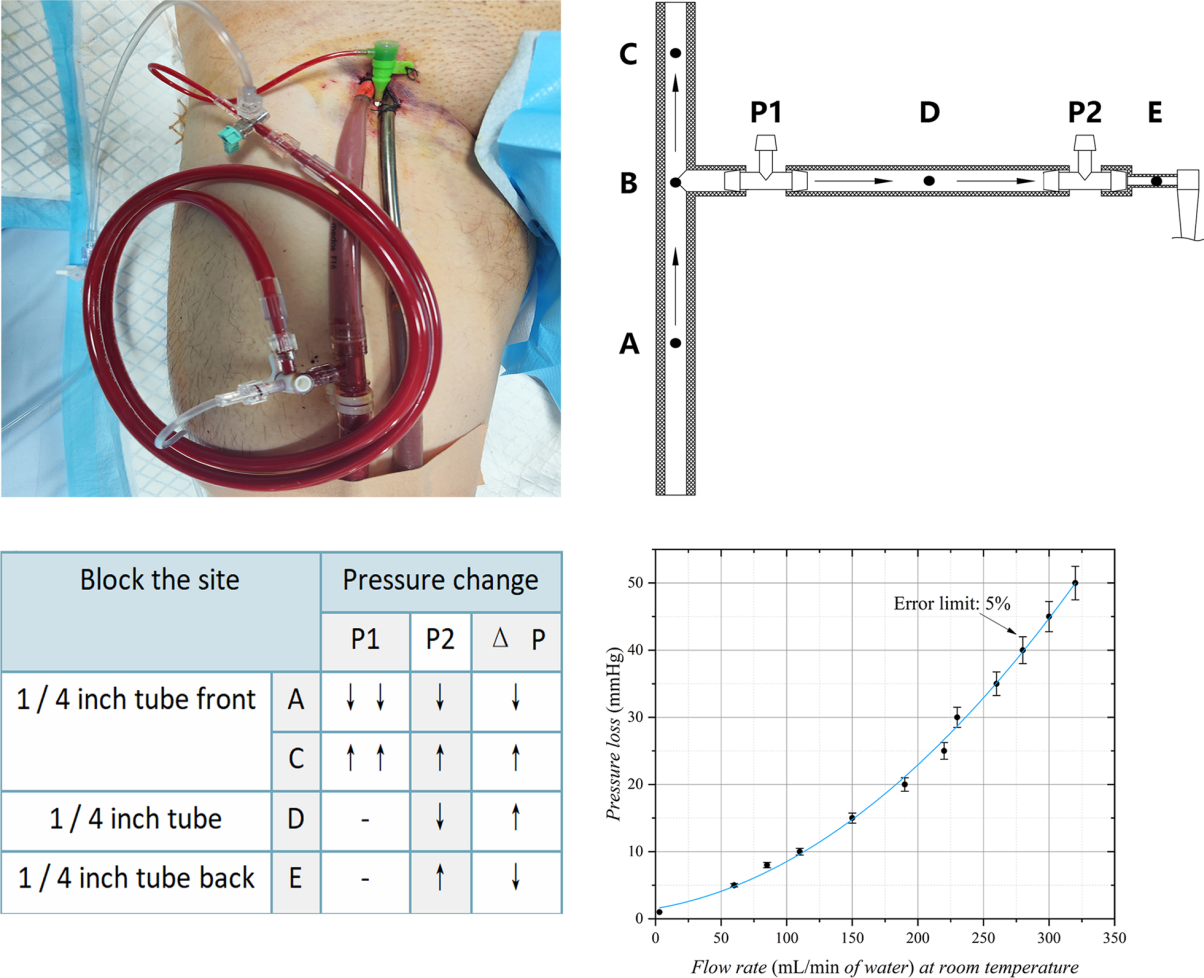
A clinically common mode of collateral connection (Left top panel); simplified clinical common connection mode (Right top panel); the blocking in different positions of the pipeline reflects the relationship of the pressure change (Left bottom panel); pressure loss flow diagram of the 1/4-inch tube (Right bottom panel)

## Data Availability

The datasets used and/or analyzed during the study are available from the corresponding author on reasonable request.

## Abbreviations

PECMO: Peripheral extracorporeal membrane oxygenation
DPC: Distal perfusion cannula
